# Predictors of Physical Frailty Transitions in Chinese Older Adults: The Role of Physical Activity and Function

**DOI:** 10.1093/geroni/igaf122.2376

**Published:** 2025-12-31

**Authors:** Ziwei Zeng, Yijian Yang

**Affiliations:** The Chinese University of Hong Kong, Hong Kong, China (People’s Republic); The Chinese University of Hong Kong, Hong Kong, China (People’s Republic)

## Abstract

Frailty is a dynamic geriatric syndrome associated with adverse health outcomes, yet its progression can be mitigated through targeted interventions. This study investigated predictors of frailty transitions in Chinese older adults, focusing on physical activity (PA) and physical performance. Using data from the China Health and Retirement Longitudinal Study (CHARLS), we examined frailty (robust, pre-frail, frail) transitions between baseline data (collected in 2011) and follow-up data (collected in 2013) among 1,014 participants aged 65 and older. Frailty was assessed using the Physical Frailty Phenotype, and PA was measured using a modified International Physical Activity Questionnaire. Physical performance was evaluated using the Short Physical Performance Battery (SPPB) and handgrip strength. Ordinal logistic regression models were used to examine the relationship between PA, physical performance, and frailty transitions. Results showed that higher PA levels and better physical performance were associated with a reduced likelihood of worsening frailty or a greater chance of transitioning to a more robust state. Among robust individuals, increased handgrip strength predicted remaining robust [Average marginal effects (AME)=1.1%, p = 0.015]. Pre-frail individuals with higher vigorous PA [AME=21.8%, p = 0.037] and handgrip strength [AME=0.6%, p = 0.003] were more likely to transition to robust state. Frail individuals with increased low-intensity PA [AME=22.5%, p = 0.038] and higher SPPB walking sub-scores [AME=27.73%, p = 0.017] had a greater probability of improving to non-frailty. These findings highlight the importance of tailored interventions based on baseline frailty status. Promoting PA and improving physical performance, particularly aspects of muscle strength and mobility, may help delay or reverse frailty progression.

